# Non-Abelian Floquet braiding and anomalous Dirac string phase in periodically driven systems

**DOI:** 10.1038/s41467-024-45302-2

**Published:** 2024-02-07

**Authors:** Robert-Jan Slager, Adrien Bouhon, F. Nur Ünal

**Affiliations:** TCM Group, Cavendish Laboratory, University of Cambridge, JJ Thomson Avenue, Cambridge, CB3 0HE United Kingdom

**Keywords:** Ultracold gases, Theoretical physics, Topological insulators

## Abstract

While a significant fraction of topological materials has been characterized using symmetry requirements^1–4^, the past two years have witnessed the rise of novel multi-gap dependent topological states^5–9^, the properties of which go beyond these approaches and are yet to be fully explored. Although already of active interest at equilibrium^10–15^, we show that the combination of out-of-equilibrium processes and multi-gap topological insights galvanize a new direction within topological phases of matter. We show that periodic driving can induce anomalous multi-gap topological properties that have no static counterpart. In particular, we identify Floquet-induced non-Abelian braiding, which in turn leads to a phase characterized by an anomalous Euler class, being the prime example of a multi-gap topological invariant. Most strikingly, we also retrieve the first example of an ‘anomalous Dirac string phase’. This gapped out-of-equilibrium phase features an unconventional Dirac string configuration that physically manifests itself via anomalous edge states on the boundary. Our results not only provide a stepping stone for the exploration of intrinsically dynamical and experimentally viable multi-gap topological phases, but also demonstrate periodic driving as a powerful way to observe these non-Abelian braiding processes notably in quantum simulators.

## Abstract (web summary)

R.-J. Slager et al. extend the theory of multigap topology from static to non-equilibrium systems. They identify Floquet-induced non-Abelian braiding, resulting in a phase characterized by anomalous Euler class, a multi-gap topological invariant. They also find a gapped anomalous Dirac string phase. Both phases have no static counterparts and exhibit distinct boundary signatures.

## Introduction

Topologically protected phases profit from connections with mathematical principles to characterize illustrious physical behavior in tangible systems^16–18^. Recent theoretical advances have led to more exotic, multi-gap topological phenomena involving non-Abelian braiding of band nodes^5–8,19^, which have importantly also been connected to observations e.g., in meta-materials^10,11,20^. The prediction of topologically stable monopole–antimonopole pairs imprinted upon quenching by a non-trivial Euler Hamiltonian^12^ has been promptly followed by experimental verification with trapped ions^13^. These rapid developments, as well as their unique non-Abelian properties, have sparked new multi-gap topological pursuits as a promising research direction in various platforms that range from phonons^14,15,21,22^ to strained electronic systems^23,24^.

Going beyond static phenomena, the study of topology in out-of-equilibrium settings has further revealed new topological classification schemes^25–29^and new connections between different invariants^30^ as signified by a vast body of work on symmetry indicated phases. In this regard, Floquet systems stand out with the periodic nature of their spectrum, where the *quasienergy* can be defined solely up to modulo 2*π*, forming the Floquet Brilloun zone (FBZ), which repeats itself in integer multiples of 2*π*. Essentially, one obtains quasienergy bands with a crucial difference, compared to static counterparts, of one additional gap at the FBZ edge, connecting the replicas of bands. With the possibility of harboring edge states also in this extra gap^26,27^, anomalous Floquet topological insulators have attracted great attention, as the Chern number has been rendered insufficient to predict edge states, also triggering experimental pursuits^31–35^.

**Fig. 1:**
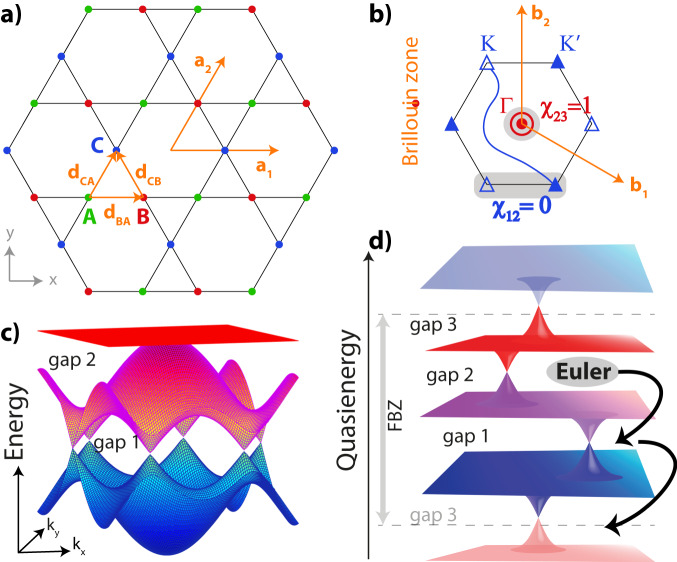
Static Kagome model and multi-gap topology in Floquet spectrum. **a** The Kagome lattice underlying model (3). **b** BZ harbors multi-gap topological configuration of band nodes visible in the static band structure (**c**) for vanishing Δ_*α*_. The double node between bands 2 and 3 (red circles) at the Γ-point is stable and characterized by patch Euler class *χ*_23_ = 1. The nodes at *K* and ${K}^{{\prime} }$ (triangles) in gap 1 are connected by a Dirac string (blue line). These nodes can be annihilated (illustrated with empty/filled markers), hence, have patch Euler class (shaded area) *χ*_12_ = 0. **d** A schematic Floquet spectrum. The periodic nature of quasienergy culminates in an extra gap (gap 3) at the edge of the Floquet Brillouin zone. As a result, non-Abelian braiding of band nodes can involve any of these band gaps.

Here, we address the fundamental question of whether multi-gap topological phases beyond equilibrium counterparts exist in Floquet settings and investigate the role of anomalous gap on the non-Abelian Euler topological classification as well as relevant physical signatures. We find that these questions can be answered affirmatively. We demonstrate novel anomalous non-Abelian phases where *all* gaps, including the anomalous Floquet gap, are required to accomplish the transition in the topological regime. In particular, band nodes can be braided between bands [which we will denote as ‘gaps’ despite being gapless due to the presence of the degeneracies] within the FBZ, as well as over the edge of the FBZ as illustrated in Fig. 1d. This similarly holds for Dirac strings which are lines that mark a change in gauge (from + to −) as will be detailed in the subsequent. We analyze Dirac strings that can reside in between bands of different FBZs and elucidate new braiding processes involving all gaps in the quasienergy spectrum, which can only occur in periodically driven systems. Moreover, we present a fully gapped phase with all nodes removed via non-Abelian braiding, while the system still hosts anomalous Dirac strings allowed by the Floquet spectrum. Importantly, these findings physically relate to unaccounted edge states, providing for a direct observable to distinguish this new anomalous phase.

## Results

### Multi-gap Floquet topology

We begin by introducing multi-gap topology and non-Abelian braiding in momentum space. Multi-gap phases acquire topological invariants in band subspaces (set of isolated bands) via multi-gap conditions and braiding of band degeneracies (band touching points). Hence, they can be independent of their irreducible representations (irreps)^2–4^ at high symmetry points and go beyond previously known topological classifications. In particular, all irreps could, in principle be trivial, a case in which the classifications of refs. ^2–4,36^ cannot discern any topology. Yet, when a system has ${{{{{{{{\mathcal{C}}}}}}}}}_{2}{{{{{{{\mathcal{T}}}}}}}}$ or ${{{{{{{\mathcal{PT}}}}}}}}$ [combination of two-fold rotations or parity and time reversal] symmetry admitting a real representation of the Hamiltonian^6–8^, the eigenstates span a real orthonormal frame. Keeping track of the rotation of the eigenvectors’ frame on a loop a band node, one then associates a frame charge corresponding to the encircled degeneracy^7^, acting as the analog of disclinations in bi-axial nematic phases^37–40^. Most interestingly, these frames can acquire a non-Abelian accumulated angle when they traverse around a node residing in another gap, resulting in non-Abelian values of the frame charge.

Braiding such *singular points* in momentum (***k***) space can thus change their signs and renders similarly-valued charged degeneracies between two bands possible. The corresponding topological obstruction to annihilate these similarly-valued charges is quantified by the Euler class. Specifically, considering the band subspace (*n*, *n* + 1) spanned by Bloch states $\left\vert {u}_{n}({{{{{{{\boldsymbol{k}}}}}}}})\right\rangle$ and $\left\vert {u}_{n+1}({{{{{{{\boldsymbol{k}}}}}}}})\right\rangle$, the reality condition can be employed to define an Euler form

1 $${{{{{{{\rm{Eu}}}}}}}}=\left\langle {\partial }_{{k}_{1}}{u}_{n}({{{{{{{\boldsymbol{k}}}}}}}})| {\partial }_{{k}_{2}}{u}_{n+1}({{{{{{{\boldsymbol{k}}}}}}}})\right\rangle -\left\langle {\partial }_{{k}_{2}}{u}_{n}({{{{{{{\boldsymbol{k}}}}}}}})| {\partial }_{{k}_{1}}{u}_{n+1}({{{{{{{\boldsymbol{k}}}}}}}})\right\rangle$$

and an associated Euler connection 1-form, ${{{{{{{\mathcal{A}}}}}}}}=\left\langle {u}_{n}({{{{{{{\boldsymbol{k}}}}}}}})| {{{{{{{\boldsymbol{\nabla }}}}}}}}{u}_{n+1}({{{{{{{\boldsymbol{k}}}}}}}})\right\rangle \cdot d{{{{{{{\boldsymbol{k}}}}}}}}$ (see Methods for a detailed exposition)^7^. When this two-band subspace is fully gapped from other bands, the integral of the non-Abelian curvature (1) over the whole BZ closely mimics the Chern number, counting the number of similarly-valued charges^12^. However, the definition remains general to situations in which the band subspace is connected via nodes to other bands, by considering a patch ${{{{{{{\mathcal{D}}}}}}}}$ in the BZ that excludes the nodes from neighboring gaps (see shaded areas in Fig. 1b). The resulting patch Euler class is evaluated by integrating over the patch upon including a boundary term over $\partial {{{{{{{\mathcal{D}}}}}}}}$,

2 $${\chi }_{n,n+1}[{{{{{{{\mathcal{D}}}}}}}}]=\frac{1}{2\pi }\left[{\int}_{{{{{{{{\mathcal{D}}}}}}}}}{{{{{{{\rm{Eu}}}}}}}}\,d{k}_{1}\wedge d{k}_{2}-{\oint }_{\partial {{{{{{{\mathcal{D}}}}}}}}}{{{{{{{\mathcal{A}}}}}}}}\right]\in {\mathbb{Z}}.$$

A particular insightful perspective to quantify the emergent multi-gap topology can be attained by considering Dirac strings that can efficiently trace non-Abelian topological phase transitions when the reality condition set by ${{{{{{{{\mathcal{C}}}}}}}}}_{2}{{{{{{{\mathcal{T}}}}}}}}$ or ${{{{{{{\mathcal{PT}}}}}}}}$ is fulfilled^11,14^, which will be useful in our characterization of Floquet systems. Each pair of nodes within a given band subspace is connected by a Dirac string (blue line in Fig. 1b). Note that these Dirac strings are gauge objects and represent the line across which the sign of the eigenstates forming a node flips upon circling around it (indicating a *π*-Berry phase of the eigenstate). When a node in the subspace of interest crosses the Dirac string of a pair of nodes in a neighboring gap, its frame charge changes sign, making it possible to obtain similarly charged band nodes in this gap. We thus recover the non-Abelian braiding characterized by the patch Euler class as defined above.

With the important role played by adjacent gaps in multi-gap topology, it is compelling to address the effect of a Floquet drive and the periodicity of the spectrum^41,42^. Under a periodic modulation with frequency *ω* = 2*π*/*T*, the Floquet spectrum is resolved as the phase eigenvalues (*e*^−*iεT*^) of the time evolution operator evaluated over a full period, $U(T)={\mathfrak{T}}\exp \{-i\int\nolimits_{0}^{T}H(t)dt\}$, for time ordering ${\mathfrak{T}}$ and the Hamiltonian *H*(*t* + *T*) = *H*(*t*). The quasinergy *ε* is defined on a circle, where we identify the FBZ with *εT* *∈* (−*π*, *π*] as marked in Fig. 1d. In the context of Euler class, we here consider periodic drives that preserve the ${{{{{{{{\mathcal{C}}}}}}}}}_{2}{{{{{{{\mathcal{T}}}}}}}}$ symmetry to ensure that the Floquet eigenstates (solutions of the time-dependent Schrödinger equation) can be brought into real form together with the non-Abelian frame charges of the band nodes^6–8^. Crucially, this renders popular circular drives inapt in common ${{{{{{{{\mathcal{C}}}}}}}}}_{2}$-symmetric setups, since the chirality breaks ${{{{{{{\mathcal{T}}}}}}}}$. As a result, two main routes emerge that either involve using linearly polarized driving or a circular drive that is rotating one direction for half a cycle and in the opposite direction for the other half.

### Model setting

To illustrate our findings, we depart from a simple Kagome geometry, given in Fig. 1, which we stress merely serves as model setting with ${{{{{{{{\mathcal{C}}}}}}}}}_{2}{{{{{{{\mathcal{T}}}}}}}}$ and in no matter affects the generality of the results presented. Taking into account nearest-neighbor (nn) hopping terms with amplitude *J* as well as on-site potentials Δ, the Hamiltonian, diagonal in crystal momentum ***k***, is written as

3 $$H({{{{{{{\boldsymbol{k}}}}}}}})=-2J\mathop{\sum}\limits_{\alpha \ne \beta }\cos ({{{{{{{\boldsymbol{k}}}}}}}}{{{{{{{{\boldsymbol{d}}}}}}}}}_{\alpha \beta }){c}_{\alpha }^{{{{\dagger}}} }{c}_{\beta }+\mathop{\sum}\limits_{\alpha }{{{\Delta }}}_{\alpha }{c}_{\alpha }^{{{{\dagger}}} }{c}_{\alpha },$$

where ${c}_{\alpha }^{({{{\dagger}}} )}$ is the annihilation (creation) operator at site *α* for (*α*, *β*) *∈* (*A*, *B*, *C*) denoting the three orbital basis, with the nn distances given by ${{{{{{{{\boldsymbol{d}}}}}}}}}_{BA}=\frac{1}{2}\hat{x},\,{{{{{{{{\boldsymbol{d}}}}}}}}}_{CA}=\frac{1}{4}\hat{x}+\frac{\sqrt{3}}{4}\hat{y}$ and ${{{{{{{{\boldsymbol{d}}}}}}}}}_{CB}=-\frac{1}{4}\hat{x}+\frac{\sqrt{3}}{4}\hat{y}$ [see Fig. 1 and Methods]. Hereafter, we set the lattice spacing to one, *a* = 1, together with the Planck’s constant and unit charge *ℏ* = *q* = 1, where energy units will be expressed in terms of *J* = 1. The BZ is defined by the reciprocal lattice vectors, ${{{{{{{{\boldsymbol{b}}}}}}}}}_{1}=2\pi \hat{x}-\frac{2\pi }{\sqrt{3}}\hat{y}$ and ${{{{{{{{\boldsymbol{b}}}}}}}}}_{2}=\frac{4\pi }{\sqrt{3}}\hat{y}$, which harbors two dispersive bands with linear band touchings in between at *K* and ${K}^{{\prime} }$ in the absence of sublattice offsets (Δ_*α*_ = 0) as shown in Fig. 1b, c. Empty/filled triangles (and other markers) represent opposite frame charges in a given gap, connected by Dirac strings. Crucially, the completely flat third band with a quadratic band touching at Γ point carries a patch Euler class *χ*_23_ = 1 between bands 2 and 3, owing to the obstruction to annihilate this double node.

We imagine a periodic modulation in the two-dimensional plane of the lattice, making the Hamiltonian (3) time-dependent, whose discrete translation symmetry can be restored by going to the frame comoving with the lattice via a gauge transformation^41,42^. Eventually, for the (effective) vector potential ${{{{{{{\boldsymbol{A}}}}}}}}(t)={A}_{x}\cos (\omega t)\hat{x}-{A}_{y}\cos (\omega t+\varphi )\hat{y}$, with *φ* controlling the polarization, the crystal momentum gets modified as ***k*** → ***k*** + ***A***(*t*) via the minimal coupling^43^. In the following, without loss of generality, we will focus on linear driving along *x*-direction with *A*_*y*_ = 0 as a route to illustrate Floquet-induced multi-gap topological phases depicted in Fig. 1d. With the tuning parameters *ω*, *A*_*x*_, Δ_*A*_ and Δ_*C*_, where we fix Δ_*B*_ = − Δ_*A*_−Δ_*C*_ for simplicity, we invoke phase transitions and evaluate the quasienergy spectra numerically together with the change of the Euler class in different gaps, see also Methods.

### Anomalous Euler phase and Floquet-induced braiding

**Fig. 2:**
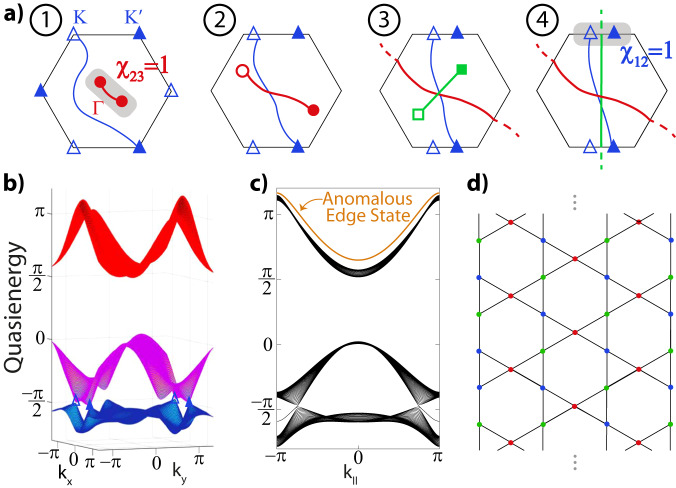
Realization of an anomalous Euler phase. **a** Starting from the Kagome model (3), linear shaking directly separates the Γ nodes (red circles, both filled as they have same charges), giving the Dirac string configuration in step 1. Subsequently, decreasing Δ_*A*_ and Δ_*C*_ (while keeping Δ_*B*_ = − Δ_*A*_−Δ_*C*_), new nodes (green boxes) in the anomalous gap are created and braided with the existing nodes as shown in steps 2–4. The green nodes eventually annihilate across the BZ, leaving behind a Dirac string and obstructing the annihilation of the $(K,{K}^{{\prime} })$ nodes (blue triangles). The Euler class is transferred from the Γ-node to the $K,{K}^{{\prime} }$ pair, as quantified by final patch Euler classes *χ*_23_ = 0 and *χ*_12_ = 1. **b** Quasienergy spectrum of the final phase (4), for Δ_*A*_ = −2.2, Δ_*C*_ = −1.5 and *A*_*x*_ = 2, *ω* = 6 in units of *J*. **c** The anomalous (green) Dirac string is incompatible with a static system, and results in an anomalous edge state for the ribbon geometry given in **d**, serving as a direct observable.

Using the outlined strategy, we now analyze the non-Abelian charges and their braiding that can be induced by periodic Floquet driving. We introduce the first anomalous Euler phase summarized in Fig. 2a, where the patch Euler class is transferred between different subspaces (nodes) by involving *all* gaps in the spectrum and, most crucially, the Dirac string (of the anomalous band nodes) in the anomalous gap at the FBZ edge.

Specifically, upon linearly driving the Kagome lattice, the Γ-nodes immediately split due to broken ${{{{{{{{\mathcal{C}}}}}}}}}_{6}$ symmetry, revealing a Dirac string (red) in between them (step 1 in Fig. 2a). We then instigate non-Abelian braiding processes by decreasing the sublattice offsets Δ_*A*_ and Δ_*C*_, allowing us to annihilate the Γ-nodes across the BZ by flipping its charge upon crossing the blue Dirac string of the ($K,{K}^{{\prime} }$) nodes. The Floquet nature comes into full play when a new pair of nodes (green squares) residing in the *π*-gap (i.e., gap 3) in between the top and bottom bands over the FBZ edge are created and separated with the green Dirac string connecting them. These anomalous band nodes still carry opposite (empty/filled) charges after crossing two Dirac strings (red, blue) of the adjacent gaps. The last step witnesses the annihilation of the anomalous (green) nodes across the BZ, ensuring their Dirac string is left behind in the middle of *K* and ${K}^{{\prime} }$. As a result, these nodes in gap 1 now have finite patch Euler class *χ*_12_ = 1 and are thus obstructed to annihilate, which we confirm by evaluating the Euler form for the final phase given in Fig. 2b for Δ_*A*_ = −2.2, Δ_*C*_ = −1.5 (see Methods for details). Consequently, this Euler phase in which the non-trivial patch Euler class has been transferred from the Γ to $(K,{K}^{{\prime} })$ nodes is indeed anomalous and does not have a static counterpart. These insights can, in fact, be corroborated by contrasting with the static system at the same offset potentials, which has a trivial Euler class for all subsets of bands where $(K,{K}^{{\prime} })$ nodes can annihilate.

We stress the anomalous nature of the retrieved phase that comes about by virtue of an intricate interplay of the Dirac string configuration, corresponding band inversions, and movement of non-Abelian charges in *all gaps*. We note that, in analogy to phase bands^44^, the gap at the edge of the FBZ can be faithfully identified by connecting to the high-frequency regime^45^ where the Floquet replicas are well separated, which has also allowed for gap-specific measurements of winding numbers in the anomalous single-gap topological context^31,45^. It might, hence, be referred to as the anomalous gap. However, a simple band inversion in this gap does not a priori result in a truly anomalous phase since the FBZ can be shifted, such that a single signature in this gap can have a static correspondence albeit with different band labeling. Instead, the anomalous Euler phase in Fig. 2 incorporates all gaps and has a final configuration featuring a Dirac string or non-Abelian Euler nodes in *each* gap, with the Dirac string in the anomalous gap being of crucial importance to induce a non-zero Euler class. These processes also profit from the constraints on the Wannier centers instigated by the Kagome lattice harboring Dirac strings already in the atomic limit, as we detail in the Methods. Moreover, the anomalous Euler phase is not only interesting from a fundamental bulk perspective but also leads to a boundary response where the synergy of the Dirac string configuration and non-zero Euler class ensures that an edge state needs to appear, see Fig. 2c, d. Namely, the band inversion generating the Dirac string in the anomalous gap induces an additional *π*-valued shift in the Zak phases. This results in the appearance of a boundary mode in the anomalous gap for surfaces to which this extra phase projects (such as in Fig. 2d), as we detail in the subsequent Methods.

We, furthermore point out that our braiding perspective also shines light on more conventional cases of dynamically inverting band spectra^41–43^. It is known that under periodic modulation, the tunneling amplitudes get renormalized by Bessel functions^42^, with the leading order captured by the zeroth-order Bessel function. Consequently, tunneling amplitudes can get dynamically frustrated (see, e.g., ref. ^46^ for experimental demonstration) or change their sign depending on the ratio of driving strength and frequency^43^. For the Kagome system, this inversion results in the flat band now being the lowest band with the sign change of *J* (see Methods for details). Our analysis indeed reveals that this naturally involves braiding of the nodes in gaps 1 and 2 upon increasing the driving amplitude. We note that in both the anomalous Euler phase introduced above and in the dynamical band inversion case, the Euler class is transferred from the nodes in gap 2 to gap 1. Despite comprising Floquet-induced braiding, however, the latter cannot be called an anomalous non-Abelian phase as these processes occur only within the FBZ. As a result, the absence of an edge state in the anomalous gap in the dynamically-inverted case provides a clear contrast with the anomalous Euler phase.

### Anomalous Dirac string phase

**Fig. 3:**
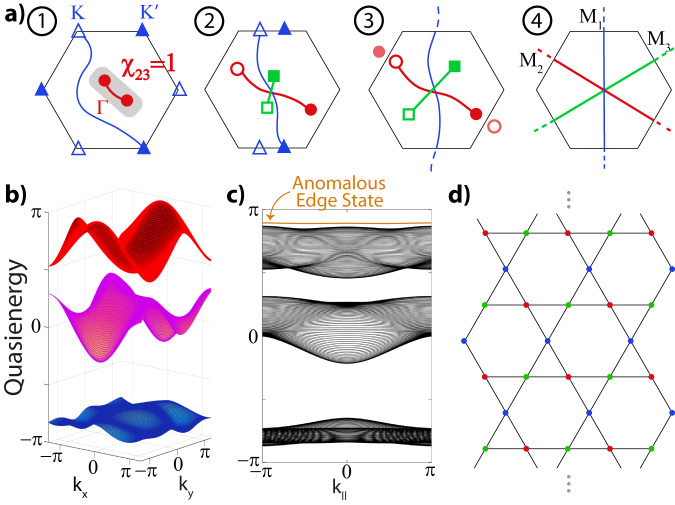
Realization of an anomalous Dirac string phase. Linearly driven Kagome model, with *ω* = 6, *A*_*x*_ = 2. **a** The multi-gap topological configuration evolves as shown in steps 1−4 upon decreasing Δ_*C*_. The final stage entails the ADS phase, a new fully gapped phase with a Dirac string in each gap, including the anomalous one. **b** Quasienergy spectrum of the ADS phase for Δ_*A*_ = 0, Δ_*C*_ = −Δ_*B*_ = −3. **c** The extra string of the anomalous gap signals an extra accumulation of *π*-Berry phase and thus an unconventional edge spectrum indicated by the relevant Zak phases, for the ribbon geometry depicted in **d**. This anomalous edge state acts as a direct observable of the ADS phase.

We showcase that novel gapped anomalous topological phases may arise by an interplay of multi-gap topological principles and the periodicity of the FBZ. Figure 3 displays an example of a new “anomalous Dirac string” (ADS) phase, which we obtain by driving with frequency *ω* = 6 and amplitude *A*_*x*_ = 2 as before while now decreasing only the offset parameter Δ_*C*_. We retrieve the familiar process of splitting the stable double Γ-node into two nodes that move along the **b**_1_-direction towards one of the *M* points, while $(K,{K}^{{\prime} })$ nodes move towards another one. Further decreasing Δ_*C*_ = − Δ_*B*_, the $(K,{K}^{{\prime} })$ nodes meet and annihilate at *M*_1_, leaving behind the blue Dirac string, while the Γ-nodes in the second gap annihilate at *M*_2_ point, creating the red Dirac string along the other direction. The anomalous nature of the phase is induced by the processes in the anomalous gap, which shows the creation of a pair of nodes that, crucially, move to the remaining *M*_3_ point at which they annihilate and leave behind the third green Dirac string. Consequently, the system ends up in a totally gapped phase with a Dirac string in each gap. As in the anomalous Euler phase, we stress that this gapped ADS phase can only exist in out-of-equilibrium Floquet settings with Dirac strings present in all gaps. The anomalous gap at the edge of the FBZ allows for the system to support this maximum number of Dirac strings, where the band nodes in each gap can annihilate at different *M* points.

As in the anomalous Euler phase, the anomalous nature of the ADS phase stems from the fact that each gap in the Floquet spectrum contributes, making it impossible to realize in a static system. We can make this more concrete. As detailed in the Methods, one can use an effective Zak phase description provided that this is done in a gap-specific manner, since similar Zak phases can give rise to different topological phases with different edge spectra that cannot be connected without going through a band inversion. We emphasize that while the Dirac strings can be moved locally, and even recombined in pairs (given that two *π*-phases sum up to zero), the absolute patch Euler class of every pair of nodes is gauge invariant. This enforces a *relative* topological stability of the Dirac string configuration over the whole (Floquet) spectrum in a given phase^14^. In this sense, Zak phases *together* with the tracking of the Dirac string configuration (the direct first homotopy charges) in each gap *does* provide for an effective description of the discovered phases, monitoring all gap-specific band inversions. Indeed, trying to shift the edge state from the anomalous gap to another gap or connecting to the trivial case, will need to proceed through band inversions, as can be seen by the incompatible nature of the Dirac string configuration with those phases. Only through band inversions the Zak phases and Dirac string configurations can be connected, underpinning the anomalous nature also quantitatively.

### Edge states of anomalous Dirac string phase

Most importantly, by effectively keeping track of the band inversions during the above-described evolution, our analysis also conveys a highlight feature of these esoteric phases, which is the appearance of anomalous edge states. While Dirac strings are gauge objects, similar to visions in lattice gauge theories, passing through a Dirac string when considering the Berry phase of non-contractible paths over the BZ does indicate a phase accumulation^47^, meaning that the Zak phase corresponding to that path shifts by *π*. Since the ADS phase is realized upon creating and then annihilating band nodes across the BZ to obtain a Dirac string in the anomalous gap, the bands on either side of the FBZ edge (bands 1 and 3) acquire an extra *π*-Zak phase^47^. As a result, edge terminations characterized by Zak phases over paths [perpendicular to this edge] that cross this string should display anomalous edge states^27^. We confirm this in a ribbon geometry presented in Fig. 3c, d, where the anomalous edge state is precisely identified in the spectrum as in the anomalous Euler phase. Namely, the Dirac string configuration of this ADS phase (similar to the anomalous Euler phase) differs from the trivial atomic limit only by the presence of the Dirac string in the anomalous gap, as demonstrated in the Methods due to the shifted Wannier centers. Hence, the anomalous gap hosts edge states that we indeed prove are completely localized, giving a universal signature of the ADS phase. This also provides a direct route to create phases with edge states in each gap.

## Discussion

We present for the first time that the recent developments culminating in novel multi-gap topological phases^5^, have anomalous counterparts that can only exist in an out-of-equilibrium setting. Specifically, we show that the anomalous gap, stemming from the time-periodic nature of the Floquet spectrum, can similarly induce non-Abelian braiding via band nodes therein, leading to Floquet-induced Euler phases. Apart from these hitherto uncharted out-of-equilibrium braiding processes, we further discover an anomalous Dirac string phase. Akin to the characteristic boundary modes of anomalous Floquet insulators, which were employed for their identification, this fully gapped ADS phase has an unconventional Dirac string configuration, resulting in a distinct edge state spectrum. These new phases thus appeal both for their non-equilibrium and multi-gap nature. Indeed, we stress that multi-gap models can systematically be formulated for a wide range of systems^5^, which, together with the applicability of periodic driving techniques^42^, furnishes various routes to realize these new anomalous phases. Here, we specifically mention trapped ion systems^13^, ultracold atoms^31,48,49^, meta-materials^11^, or Floquet engineering of real materials^50,51^. We, therefore, anticipate that our results form a stepping stone for the theoretical investigation of new exotic topologies and their experimental observation^52^.

## Methods

### Euler class and multi-gap topological characterizations

To classify wave functions over a Brillouin zone or Floquet bands over a Floquet Brillouin zone from a topological perspective, one is essentially interested in characterizing the describing vector bundle. Although there has been a wealth of results on getting different topological invariants, we focus on new developments that take into account multi-gap conditions^5^. We recall that oriented real bundles over a base space *B* admit a characterizing Euler class, being an element of the de Rham cohomolgy. More specifically, integrating the Euler class^7,53,54^, over a base space with no boundary results in integer in units of 2*π*, meaning that the Euler form integral defines an element of the singular chomology with integer coefficients ${H}^{2}(B,{\mathbb{Z}})$. From a physical perspective, it has been shown that the Euler class of a two-band isolated subspace directly conveys the stability of the nodes in that subspace. When a system enjoys ${{{{{{{{\mathcal{C}}}}}}}}}_{2}{{{{{{{\mathcal{T}}}}}}}}$ or ${{{{{{{\mathcal{P}}}}}}}}{{{{{{{\mathcal{T}}}}}}}}$ symmetry, band degeneracies in adjacent gaps, that is, in the gap situated directly next to the gap under consideration, carry non-Abelian frame charges^6–8^. For example, when one considers a three-band system, the reality condition allows for a frame or dreibein interpretation, and the band nodes act like *π*-vortices, whose frame charges take values in the quaternion group^7^, similar to how disclinations in bi-axial nematic phases can carry quaternionin charges^37,39,40^. From a more mathematical point of view, this can be directly seen from the fact that the describing Flag variety relates to SO(3)/D_2_^5–7^. The orientation of the frame or dreibein needs to be fixed^5,12^, ensuring that multiplying any eigenvector spanning the frame with a minus sign is a priori a gauge degree of freedom, rendering the mentioned SO(3)/D_2_ parametrization. The first homotopy group ${\pi }_{1}[{\mathsf{SO}}(3)/{{\mathsf{D}}}_{2}]={\mathbb{Q}}=\{+1,\pm i,\pm j,\pm k,-1\}$ accordingly reveals the frame charges. The Euler class of the gapped isolated two-band subspace then physically pertains to the stability of the nodes, giving a finite value when the frame charges of the nodes do not add to zero^7^.

Most interestingly, the Euler class can be refined to a patch Euler class *χ*, which essentially evaluates the Euler class over a patch in the Brillouin zone, taking into account a boundary term^7^. The explicit expression for a two-band subspace spanned by bands ${{{{{{{{\mathcal{B}}}}}}}}}_{n}$ and ${{{{{{{{\mathcal{B}}}}}}}}}_{n+1}$ reads

4 $${\chi }_{n,n+1}[{{{{{{{\mathcal{D}}}}}}}}]\equiv \chi [\{{{{{{{{{\mathcal{B}}}}}}}}}_{n},{{{{{{{{\mathcal{B}}}}}}}}}_{n+1}\};{{{{{{{\mathcal{D}}}}}}}}]=\frac{1}{2\pi }\left[{\int}_{{{{{{{{\mathcal{D}}}}}}}}}{{{{{{{\rm{Eu}}}}}}}}-{\oint }_{\partial {{{{{{{\mathcal{D}}}}}}}}}{{{{{{{\rm{a}}}}}}}}\right]\in {\mathbb{Z}},$$

where the Euler 2-form ‘Eu’ is integrated over the patch ${{{{{{{\mathcal{D}}}}}}}}$ and supplemented with a boundary term that integrates the Euler connection 1-form ‘a’ over the contour $\partial {{{{{{{\mathcal{D}}}}}}}}$ of the patch. The Euler 2-form in the above expression in terms of the wave function *u*_*a*_ is defined as ${{{{{{{\rm{Eu}}}}}}}}=d{{{{{{{\rm{a}}}}}}}}=d{{{{{{{\rm{Pf}}}}}}}}{{{{{{{\mathcal{A}}}}}}}}$ with ${{{{{{{{\mathcal{A}}}}}}}}}_{ab}=\langle {u}_{a},{{{{{{{\boldsymbol{k}}}}}}}}| d{u}_{b},{{{{{{{\boldsymbol{k}}}}}}}}\rangle={{{{{{{{\boldsymbol{A}}}}}}}}}_{ab}\cdot d{{{{{{{\boldsymbol{k}}}}}}}}={\sum }_{i=1,2}\langle {u}_{a},{{{{{{{\boldsymbol{k}}}}}}}}| {\partial }_{{k}_{i}}{u}_{b},{{{{{{{\boldsymbol{k}}}}}}}}\rangle d{k}_{i}$ where we have set ${A}_{ab}^{i}=\langle {u}_{a},{{{{{{{\boldsymbol{k}}}}}}}}| {\partial }_{{k}_{i}}{u}_{b},{{{{{{{\boldsymbol{k}}}}}}}}\rangle$ (*A*^*i*^ *∈* SO(2)). This gives ${{{{{{{\rm{Eu}}}}}}}}=(\langle {\partial }_{{k}_{1}}{u}_{a},{{{{{{{\boldsymbol{k}}}}}}}}| {\partial }_{{k}_{2}}{u}_{b},{{{{{{{\boldsymbol{k}}}}}}}}\rangle - \langle {\partial }_{{k}_{2}}{u}_{a},{{{{{{{\boldsymbol{k}}}}}}}}| {\partial }_{{k}_{1}}{u}_{b},{{{{{{{\boldsymbol{k}}}}}}}}\rangle )d{k}_{1}\wedge d{k}_{2}$, where *a*, *b* take values in the band indices *n*, *n* + 1^7,8^. In addition, the Euler connection 1-form is given as a = Pf***A*** ⋅ *d****k***.

The above characterization assumes a real bundle structure. In other words, we need to maintain either ${{{{{{{{\mathcal{C}}}}}}}}}_{2}{{{{{{{\mathcal{T}}}}}}}}$ or ${{{{{{{\mathcal{P}}}}}}}}{{{{{{{\mathcal{T}}}}}}}}$ symmetry to ensure that the Hamiltonian and wave functions admit a real formulation. Focusing on Floquet systems the constraint to have real eigenvectors *u*_*a*_ restricts the type of the periodic modulation. Hence, we focus on linearly polarized driving as they can preserve time-reversal symmetry in most common ${{{{{{{{\mathcal{C}}}}}}}}}_{2}$-symmetric setups, fulfilling the reality conditions.

We start from the real-space tight-binding Hamiltonian, $H=-J{\sum }_{j{j}^{{\prime} }}{c}_{j}^{{{{\dagger}}} }{c}_{{j}^{{\prime} }}$, on the Kagome lattice, where $\{j,{j}^{{\prime} }\}$ run through all nearest-neighbor pairs. We consider a periodic driving which induces a force, ${{{{{{{\bf{F}}}}}}}}(t)={F}_{x}\sin (\omega t)\hat{x}+{F}_{y}\sin (\omega t+\varphi )\hat{y}$ on the lattice site **r**_*j*_, making the Hamiltonian time dependent, $H(t)=H-{\sum }_{j}{{{{{{{\bf{F}}}}}}}}(t)\cdot{{{{{{{{\bf{r}}}}}}}}}_{j}\,{c}_{j}^{{{{\dagger}}} }{c}_{j}$. After performing a gauge transformation, $R=\exp \{-i{\sum }_{j}\int\,dt{{{{{{{\bf{F}}}}}}}}(t)\cdot\,{{{{{{{{\bf{r}}}}}}}}}_{j}\,{c}_{j}^{{{{\dagger}}} }{c}_{j}\}$, to the frame of the lattice^42,49^, we obtain a simple form of the Hamiltonian in which the tunneling amplitudes are modified with the Peierls substitution ${J}_{j{j}^{{\prime} }}(t)=J\exp \{-i\int\nolimits_{{{{{{{{{\bf{r}}}}}}}}}_{{j}^{{\prime} }}}^{{{{{{{{{\bf{r}}}}}}}}}_{j}}{{{{{{{\bf{A}}}}}}}}(t)\cdot d{{{{{{{\bf{r}}}}}}}}\}$, where we define the effective vector potential ${{{{{{{\boldsymbol{A}}}}}}}}(t)={A}_{x}\cos (\omega t)\hat{x}-{A}_{y}\cos (\omega t+\varphi )\hat{y}$ with *A*_*i*_ = *F*_*i*_/*ω* [the Planck’s constant and lattice spacing are set to one for simplicity]. Hence, we control the strength of our periodic modulation by tuning (*A*_*x*_, *A*_*y*_) and, in this work, focus on linear driving only along *x*-direction by setting *A*_*y*_ = 0. Eventually, this modifies the momentum space Hamiltonian in Eq. (3) under minimal coupling to $H({{{{{{{\boldsymbol{k}}}}}}}},t)=-2J{\sum }_{\alpha \ne \beta }\cos [({{{{{{{\boldsymbol{k}}}}}}}}+{{{{{{{\bf{A}}}}}}}}(t))\cdot {{{{{{{{\bf{d}}}}}}}}}_{\alpha \beta }]{c}_{\alpha }^{{{{\dagger}}} }{c}_{\beta }+{\sum }_{\alpha }{{{\Delta }}}_{\alpha }{c}_{\alpha }^{{{{\dagger}}} }{c}_{\alpha }$^43^. We numerically calculate the time evolution operator at the end of one full period of the drive, $U({{{{{{{\boldsymbol{k}}}}}}}},T)={\mathfrak{T}}\exp \{-i\int\nolimits_{0}^{T}H({{{{{{{\boldsymbol{k}}}}}}}},t)dt\}$ and evaluate the quasienergy *ε*(***k***) as its phase eigenvalues, *e*^−*iε*(***k***)*T*^, which can be defined only up to modulo 2*π*.

### Characterizing the anomalous Euler phase

With the above characterization of Euler class and multi-gap topology in terms of wave functions, we may directly analyze band spaces of Floquet systems. We note that, in fact multi-gap conditions arise naturally as there is no fixed band ordering since the Floquet spectrum repeats itself in multiples of the driving frequency.

**Fig. 4:**
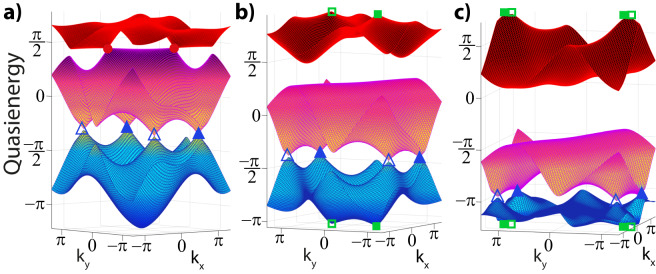
Floquet spectra of intermediate stages to reach the anomalous Euler phase. Band structures during the evolution depicted in Fig. 2 show the anomalous braiding process for frequency *ω* = 6 and amplitude *A*_*x*_ = 2. The eigenvectors directly characterize the Euler class and quantify the braiding. The offset parameters [Δ_*A*_, Δ_*C*_] (where Δ_*B*_ = − Δ_*A*_−Δ_*C*_) are tuned as **a**) [−0.5, −0.5], **b** [−1, −1] and **c** [−1.9, −1.5], right before the anomalous (green) nodes annihilate to give rise Fig. 2b.

Departing from the static regime^11^, we follow the progression of the bands as we tune the parameters of the system, see Fig. 2 of the main text. This achieves a braiding using the nodes of the anomalous gap over the Floquet Brillouin zone, which can be quantified using the patch Euler class of Eq. (2), profiting from the discussed real gauge. For completeness, we demonstrate the evolution and the braiding of the band nodes in Fig. 4, which also includes the anomalous band nodes (green) between bands 1 and 3. We confirm these braiding processes by numerically calculating the patch Euler class of the final anomalous Euler phase given in Fig. 2.

It is instructive to further underpin the truly anomalous nature of the described Euler phase by comparing to its static counterpart with the same sublattice potentials. We stress that, in this case, the band structure shows a trivial multi-gap topological configuration. Indeed, the nodes between the first and second bands can be gapped, which is corroborated by a calculation of the Euler patch class that is trivial for any patch for the nodal intermediate regions until the band structure is fully gapped.

**Fig. 5:**
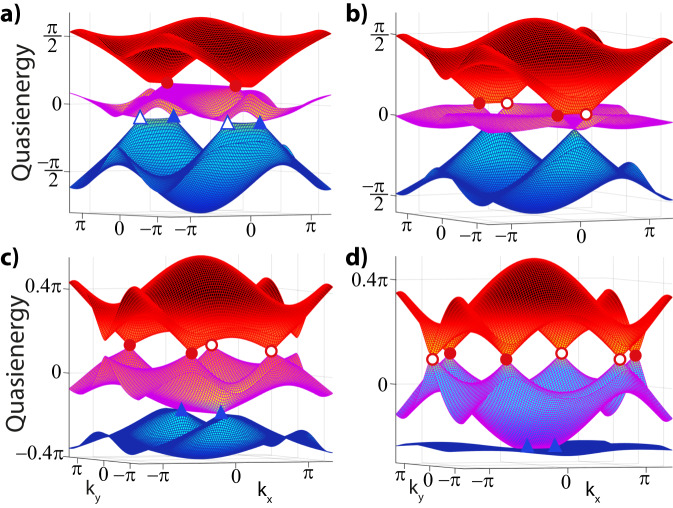
Floquet non-Abelian braiding and inverted spectrum. Starting from the static Kagome model, Euler charge is transferred from gap 2 to gap 1 by increasing the driving strength at fixed frequency *ω* = 6 and vanishing sublattice offsets Δ_*A*_ = Δ_*B*_ = Δ_*C*_ = 0. The driving strength *A*_*x*_ is varied to take values **a** 4, **b** 5, **c** 6, and **d** 7.

**Fig. 6:**
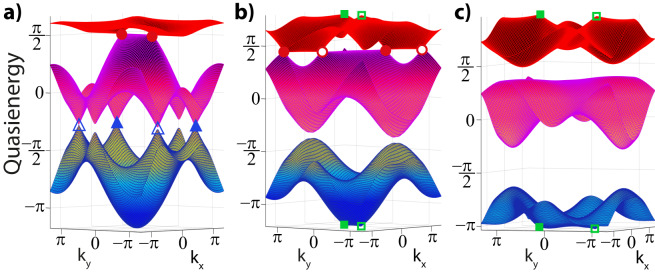
Floquet band structures of intermediate stages to reach the Anomalous Dirac string phase. **a** Driving [here with frequency *ω* = 6, and amplitude *A*_*x*_ = 2] splits the double node at the Γ point. As detailed in the main text, the ADS phase comes into existence by decreasing Δ_*C*_ = − Δ_*B*_ (at fixed Δ_*A*_ = 0). Successively, the $(K,{K}^{{\prime} })$ nodes between band 2 and 3 (blue triangles) and the Γ-nodes (red circles) between bands 1 and 2 annihilate and leave behind Dirac strings shown in Fig. 3a. Similarly, nodes in the anomalous gap (green boxes) are created and annihilated across the BZ, giving rise to the anomalous Dirac string. The panels show snapshots of the evolution to reach the final stage in Fig. 3b, for **a** Δ_*C*_ = 0, **b** Δ_*C*_ = −1.2, and **c** Δ_*C*_ = −2.

Secondly, we present another example of Floquet-induced braiding in Fig. 5, which we now attain by tuning the driving strength while keeping the sublattice offsets at zero, hence, also connecting to the case of dynamically inverting the bands. It is known that the tunneling amplitudes can be frustrated^46^ or made to change sign^43^ by tuning the driving strength. This effect can be understood by looking at effective time-independent tunneling amplitudes at leading order, ${J}_{{{\mbox{eff}}}}=J{{{{{{{{\mathcal{J}}}}}}}}}_{0}(A)$^42^, which gets normalized by the zeroth-order Bessel function ${{{{{{{{\mathcal{J}}}}}}}}}_{0}$ that can indeed vanish or become negative as a function of *A*. Therefore, one can obtain a spectrum with the flat band with the non-trivial Euler patch class node at the bottom for large *A*, which requires to be addressed from a braiding perspective. As discussed in the main text, linearly driving the Kagome lattice breaks ${{{{{{{{\mathcal{C}}}}}}}}}_{6}$ symmetry and separates the Γ-nodes, as shown in Fig. 6a for *A*_*x*_ = 2. Upon further increasing the driving strength, our analysis reveals that the Γ nodes and $K,{K}^{{\prime} }$ nodes move towards the same *M* point, where the latter is shown to touch in Fig. 5b for *A*_*x*_ = 5. However, instead of annihilating each other and gapping out, the system undergoes rearrangement of Dirac strings where now the Γ nodes in gap 2 carry opposite charges while the nodes in gap 1 are the same valued. This, thus effectively amounts to a transfer of Euler charge and the inversion of the band spectrum where the flat band is situated at the bottom. Further increasing the driving strength, we indeed observe that these nodes now move along directions perpendicular to their previous movements and arrange themselves according to the inverted spectrum associated with effective tunneling amplitudes with opposite sign. We stress, however that the essential dynamics are captured by a multi-gap braiding perspective, addressing also the topological stability of the $(K,{K}^{{\prime} })$ nodes illustrated with filled triangles in Fig. 5d.

### Characterization of anomalous Dirac string phase

Analogous to the Floquet-induced braiding process culminating in the anomalous Euler phase, we can further concretize the Dirac string phase in the outlined model setting, although we stress the generality of these phases. Accordingly, in Fig. 6 we present the band structures of the different stages as shown in Fig. 3a. A Dirac string physically connects band nodes^11^ and thus can be directly tracked by examining the evolution of the bands.

**Fig. 7:**
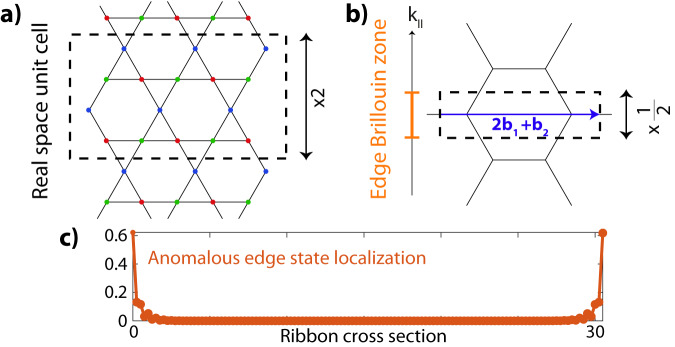
Edge Brillouin zone determination for ‘arm chair’ cut. **a** The real-space ribbon geometry of the main text. The unit cell is doubled along the periodic direction, which results in a halved-edge Brillouin zone illustrated in orange (**b**). The edge states for a band ${{{{{{{\mathcal{B}}}}}}}}$ are determined by Zak phases ${\gamma }_{{{{{{{{\mathcal{B}}}}}}}}}[2{{{{{{{{\bf{b}}}}}}}}}_{1}+{{{{{{{{\bf{b}}}}}}}}}_{2}]$ along paths perpendicular to this edge. **c** Real-space localization of the two anomalous edge states *ψ*_1_(*x*, *k*_∥_) and *ψ*_2_(*x*, *k*_∥_) in the ADS phase as function of *x* along the ribbon cross-section. Due to the difference of the Zak phase configuration in the ADS phase with respect to the atomic limit, these edge states show characteristic real-space localization, $\frac{1}{N}\mathop{\sum }\nolimits_{{k}_{\parallel }}^{N}{\sum }_{y}(| {\psi }_{1}(x,y,{k}_{\parallel }){| }^{2}+| {\psi }_{2}(x,y,{k}_{\parallel }){| }^{2})$, where we considered *N* = 30 momentum points in the edge BZ and *y* runs over the sites in the unit cell.

**Fig. 8:**
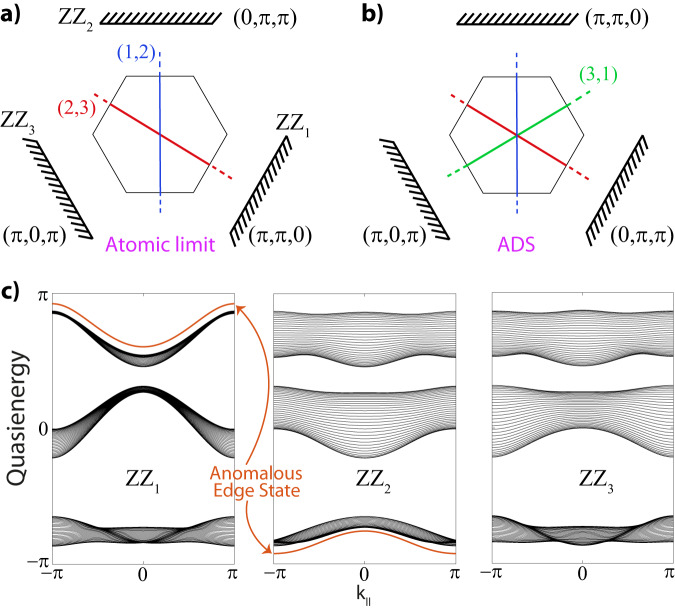
Zigzag edges and Dirac strings in atomic limit and ADS phase. **a** Dirac string configuration of the atomic limit defined in text. The three zigzag edges are characterized by the Zak phases over paths along the perpendicular directions in momentum space. *ZZ*_1_, the edge perpendicular to **b**_1_, results in $({\gamma }_{{{{{{{{{\mathcal{B}}}}}}}}}_{1}}^{AL}[{{{{{{{{\bf{b}}}}}}}}}_{1}],\,{\gamma }_{{{{{{{{{\mathcal{B}}}}}}}}}_{2}}^{AL}[{{{{{{{{\bf{b}}}}}}}}}_{1}],\,{\gamma }_{{{{{{{{{\mathcal{B}}}}}}}}}_{3}}^{AL}[{{{{{{{{\bf{b}}}}}}}}}_{1}])=(\pi,\pi,0)$, whereas *ZZ*_2_ and *ZZ*_3_ are characterized by $({\gamma }_{{{{{{{{{\mathcal{B}}}}}}}}}_{1}}^{AL}[{{{{{{{{\bf{b}}}}}}}}}_{2}],\,{\gamma }_{{{{{{{{{\mathcal{B}}}}}}}}}_{2}}^{AL}[{{{{{{{{\bf{b}}}}}}}}}_{2}],\,{\gamma }_{{{{{{{{{\mathcal{B}}}}}}}}}_{3}}^{AL}[{{{{{{{{\bf{b}}}}}}}}}_{2}])=(\pi,0,\pi )$ and $({\gamma }_{{{{{{{{{\mathcal{B}}}}}}}}}_{1}}^{AL}[{{{{{{{\bf{v}}}}}}}}=-({{{{{{{{\bf{b}}}}}}}}}_{1}-{{{{{{{{\bf{b}}}}}}}}}_{2})],\,{\gamma }_{{{{{{{{{\mathcal{B}}}}}}}}}_{2}}^{AL}[{{{{{{{\bf{v}}}}}}}}],\,{\gamma }_{{{{{{{{{\mathcal{B}}}}}}}}}_{3}}^{AL}[{{{{{{{\bf{v}}}}}}}}])=(0,\pi,\pi )$, respectively. **b** Same edge terminations and relevant Zak phases for the ADS phase, which differs from the atomic limit by the presence of a Dirac string in the anomalous gap. **c** Edge state spectra for the zigzag edges in the ADS phase. The *ZZ*_1_ and *ZZ*_2_ terminations show anomalous edge states due to the difference in Zak phases with respect to the atomic limit, while *ZZ*_3_ has no edge states.

We can further corroborate the Dirac string configuration by evaluating the Zak phases of the bands and edge states, see Fig. 7, where we recall that a Zak phase is obtained by integrating the Berry phase over a non-contractible path in the Brillouin zone^47,55^. As discussed in the main text, when a Dirac string resides between two bands, it indicates a phase accumulation of *π* in Berry phase for each crossing of the path with the string. Specifically, turning to the Dirac string configuration of the ADS phase discussed the main text; see also Figs. 3, 7 and 8b), we have Dirac strings between each band which we will denote as DS_1,2_, DS_2,3_, and DS_3,1_. Here, the subscripts refer to the bands which we number from the lowest to the highest by keeping track of their labeling during the evolution from the static Kagome limit. Hence, DS_1,3_ corresponds to the Dirac string in the anomalous gap.

We close the circle of our analysis by relating the Dirac string configuration and the Zak phases of the bands. Given the band inversion processes [Fig. 6] of the various stages described in the main text that lead to the ADS phase, we obtain the string configuration presented in Fig. 8b) where the band nodes at each gap are annihilated at a different *M* point. We can thus readily infer the Zak phase ${\gamma }_{{{{{{{{{\mathcal{B}}}}}}}}}_{1}}$ of band 1 along the non-contractible path **b**_1_. As this path crosses DS_1,2_ and DS_3,1_, but not DS_2,3_, we find ${\gamma }_{{{{{{{{{\mathcal{B}}}}}}}}}_{1}}[{{{{{{{{\bf{b}}}}}}}}}_{1}]=0$. This is because band 1 acquires a phase factor *π* from both DS_1,2_ and DS_3,1_. Similar reasoning shows that ${\gamma }_{{{{{{{{{\mathcal{B}}}}}}}}}_{2}}[{{{{{{{{\bf{b}}}}}}}}}_{1}]=\pi$ and ${\gamma }_{{{{{{{{{\mathcal{B}}}}}}}}}_{3}}[{{{{{{{{\bf{b}}}}}}}}}_{1}]=\pi$, as these Zak phases get a single *π*-contribution from DS_1,2_ and DS_1,3_, respectively. The same procedure can also be repeated for the path along **b**_2_, giving that $({\gamma }_{{{{{{{{{\mathcal{B}}}}}}}}}_{1}}[{{{{{{{{\bf{b}}}}}}}}}_{2}],{\gamma }_{{{{{{{{{\mathcal{B}}}}}}}}}_{2}}[{{{{{{{{\bf{b}}}}}}}}}_{2}],{\gamma }_{{{{{{{{{\mathcal{B}}}}}}}}}_{3}}[{{{{{{{{\bf{b}}}}}}}}}_{2}])=(\pi,\pi,0)$. Upon numerically calculating the Zak phases of the bands in the ADS regime, we indeed verify these insights and Dirac strings.

### Edge state counting in anomalous Dirac string phase

An important physical consequence that characterizes the anomalous Dirac string (ADS) phase is the appearance of edge states. As we highlighted in the main text, the transition to the ADS phase is characterized by the formation of nodes, that leave behind a Dirac string. While tracking the spectral evolution is effective in characterizing the topological phases, we here would like to further quantify the edge state spectra, using these intricate relations between the Zak phases and the various Dirac string configurations.

In static systems, a Zak phase of *π*(0) indicates the presence (absence) of edge states when the orbitals in real space are centered at the maximally symmetric Wyckoff position^47,55,56^. When the orbitals are “shifted”, corresponding to the boundary of the unit cell as in the case of our Kagome system, the role of the 0 and *π*-Zak phases is interchanged, and a *π*-Berry phase corresponds to having no edge states. Essentially, the mismatch may be quantified by counting the difference in charges of bands and Wannier centers as formalized by charge anomalies^56^. It is tempting to directly infer from the Zak phases whether an edge termination will give edge states or not in the Floquet setting. Here diligence, however, has to be taken with the out-of-equilibrium nature of the system. Indeed, due to the time periodicity of the Floquet Brillouin zone, the system is not simply adiabatically connected to the static counterpart, as *each* band now relates to gaps above and below. As an example, we consider starting from a static system where the Zak phase configuration of the three bands relevant for a certain edge projection reads $({\gamma }_{{{{{{{{{\mathcal{B}}}}}}}}}_{1}},{\gamma }_{{{{{{{{{\mathcal{B}}}}}}}}}_{2}},{\gamma }_{{{{{{{{{\mathcal{B}}}}}}}}}_{3}})=(0,\pi,\pi )$. Let us further assume that this configuration entails an atomic limit giving no edge states. Upon going to the Floquet counterpart, we may consider driving a band inversion in the anomalous gap, inducing a Dirac string and thus a *π* shift in the Zak phase of those bands. This then results in the configuration $({\gamma }_{{{{{{{{{\mathcal{B}}}}}}}}}_{1}},{\gamma }_{{{{{{{{{\mathcal{B}}}}}}}}}_{2}},{\gamma }_{{{{{{{{{\mathcal{B}}}}}}}}}_{3}})=(\pi,\pi,0)$ and an anticipated edge state in the anomalous gap. However, starting from the same initial configuration we may also consider inducing first a band inversion between the two top bands and then the two bottom bands successively. In this scenario, we thus anticipate edge states between bands 1 and 2 and bands 2 and 3. Nonetheless, in this case, the Berry phases also amount to $({\gamma }_{{{{{{{{{\mathcal{B}}}}}}}}}_{1}},{\gamma }_{{{{{{{{{\mathcal{B}}}}}}}}}_{2}},{\gamma }_{{{{{{{{{\mathcal{B}}}}}}}}}_{3}})=(\pi,\pi,0)$, showing the subtly of predicting edge states. Indeed, rather than focusing solely on the Berry phases, one in fact needs to keep track of the Berry phases, Dirac string *and* the band evolution.

The above analysis shows that we have to start from a universal description in a well-defined limit, a requirement that is unequivocally set by a trivial *atomic limit* (AL). To this end, we consider Hamiltonian (3) with all hopping terms switched off, i.e., *J* = 0, and on-site potentials (Δ_*A*_, Δ_*B*_, Δ_*C*_) = (0, 1, −1). This evidently realizes a trivially gapped system where the top, middle and bottom band corresponds to the localized wave functions on the *B*, *A,* and *C* cites, respectively. As the Wannier centers are not localized at the center, this phase does have a Dirac string configuration, as presented in Fig. 8a). Characterizing the real-space positions of the orbitals *A*, *B,* and *C* as **r**_*A*,*B*,*C*_, a simple calculation then indeed corroborates that

$$({\gamma }_{{{{{{{{{\mathcal{B}}}}}}}}}_{1}}^{AL}[{{{{{{{{\bf{b}}}}}}}}}_{1}],{\gamma }_{{{{{{{{{\mathcal{B}}}}}}}}}_{2}}^{AL}[{{{{{{{{\bf{b}}}}}}}}}_{1}],{\gamma }_{{{{{{{{{\mathcal{B}}}}}}}}}_{3}}^{AL}[{{{{{{{{\bf{b}}}}}}}}}_{1}])=	({e}^{i{{{{{{{{\bf{r}}}}}}}}}_{C}\cdot {{{{{{{{\bf{b}}}}}}}}}_{1}},\,{e}^{i{{{{{{{{\bf{r}}}}}}}}}_{A}\cdot {{{{{{{{\bf{b}}}}}}}}}_{1}},\,{e}^{i{{{{{{{{\bf{r}}}}}}}}}_{B}\cdot {{{{{{{{\bf{b}}}}}}}}}_{1}})\\=	(\pi,\pi,0),$$

for the energy-determined band ordering ${{{{{{{{\mathcal{B}}}}}}}}}_{1} \, < \, {{{{{{{{\mathcal{B}}}}}}}}}_{2} \, < \, {{{{{{{{\mathcal{B}}}}}}}}}_{3}$. The Zak phases along the other direction are readily verified to amount to $({\gamma }_{{{{{{{{{\mathcal{B}}}}}}}}}_{1}}^{AL}[{{{{{{{{\bf{b}}}}}}}}}_{2}],\,{\gamma }_{{{{{{{{{\mathcal{B}}}}}}}}}_{2}}^{AL}[{{{{{{{{\bf{b}}}}}}}}}_{2}],\,{\gamma }_{{{{{{{{{\mathcal{B}}}}}}}}}_{3}}^{AL}[{{{{{{{{\bf{b}}}}}}}}}_{2}])=(0,\pi,\pi )$. A simple analysis similar to the one presented in the previous subsection then indeed shows that the Dirac string configuration of the AL phase presented in Fig. 8a is consistent with these Zak phases.

With the trivial reference state in place, we can now effectively characterize the edge state spectrum for the “arm chair" termination studied in the main text, in a more concrete manner that corroborates the results of tracking the band inversion processes. In Fig. 7, we show the ribbon termination and the corresponding momentum space description. The spectrum should be analyzed by the Zak phases that project to the edge Brillouin zone. We observe that for this edge geometry, the unit cell is effectively doubled along the periodic direction and hence, half as large in reciprocal space. Concretely, this results in edge states which can be directly observed^57^. These relate to the Zak phases along the path 2**b**_1_ + **b**_2_ perpendicular to the vertical edge. Considering the trivial AL reference phase, it is easy to see that we obtain $({\gamma }_{{{{{{{{{\mathcal{B}}}}}}}}}_{1}}^{AL}[2{{{{{{{{\bf{b}}}}}}}}}_{1}+{{{{{{{{\bf{b}}}}}}}}}_{2}],\,{\gamma }_{{{{{{{{{\mathcal{B}}}}}}}}}_{2}}^{AL}[2{{{{{{{{\bf{b}}}}}}}}}_{1}+{{{{{{{{\bf{b}}}}}}}}}_{2}],\,{\gamma }_{{{{{{{{{\mathcal{B}}}}}}}}}_{3}}^{AL}[2{{{{{{{{\bf{b}}}}}}}}}_{1}+{{{{{{{{\bf{b}}}}}}}}}_{2}])=(0,\pi,\pi )$, as the factor 2 trivializes any contribution from **b**_1_, hence ensuring the result is the same as considering the path along **b**_2_. Comparing subsequently to the anomalous Dirac string (ADS) phase, we observe that the extra Dirac string induces another *π*-phase for the top and bottom band, $({\gamma }_{{{{{{{{{\mathcal{B}}}}}}}}}_{1}}^{ADS}[2{{{{{{{{\bf{b}}}}}}}}}_{1}+{{{{{{{{\bf{b}}}}}}}}}_{2}],\,{\gamma }_{{{{{{{{{\mathcal{B}}}}}}}}}_{2}}^{ADS}[2{{{{{{{{\bf{b}}}}}}}}}_{1}+{{{{{{{{\bf{b}}}}}}}}}_{2}],\,{\gamma }_{{{{{{{{{\mathcal{B}}}}}}}}}_{3}}^{ADS}[2{{{{{{{{\bf{b}}}}}}}}}_{1}+{{{{{{{{\bf{b}}}}}}}}}_{2}])=(\pi,\pi,0)$.

We can thus imagine starting from the atomic limit as defined above and inducing driving without closing any of the gaps. This phase should then evidently have no edge states. As a next step, the anomalous Dirac string is entered upon a band inversion in the anomalous gap, and edge states are anticipated within that gap, as consistent with the observation in Fig. 3 of the main text and quantified via the Zak phase configuration. We reemphasize that the order of processes in the band evolution needs to be taken into account to interpret these indices.

The above-presented analysis can be employed for any type of edge termination. Hence, to conclude, we comment on the edge in the three main “zigzag” directions demonstrated in Fig. 8a, b, where we cut the BZ in three directions that we label as *ZZ*_1_, *ZZ*_2_, and *ZZ*_3_. By applying the similar analysis that we employed in the study of the previous edge termination, we obtain that in the *ZZ*_1_-cut the Zak phase configuration reads $({\gamma }_{{{{{{{{{\mathcal{B}}}}}}}}}_{1}}^{AL}[{{{{{{{{\bf{b}}}}}}}}}_{1}],\,{\gamma }_{{{{{{{{{\mathcal{B}}}}}}}}}_{2}}^{AL}[{{{{{{{{\bf{b}}}}}}}}}_{1}],\,{\gamma }_{{{{{{{{{\mathcal{B}}}}}}}}}_{3}}^{AL}[{{{{{{{{\bf{b}}}}}}}}}_{1}])=(\pi,\pi,0)$. Evaluating the configuration in the ADS phase, we get $({\gamma }_{{{{{{{{{\mathcal{B}}}}}}}}}_{1}}^{ADS}[{{{{{{{{\bf{b}}}}}}}}}_{1}],\,{\gamma }_{{{{{{{{{\mathcal{B}}}}}}}}}_{2}}^{ADS}[{{{{{{{{\bf{b}}}}}}}}}_{1}],\,{\gamma }_{{{{{{{{{\mathcal{B}}}}}}}}}_{3}}^{ADS}[{{{{{{{{\bf{b}}}}}}}}}_{1}])=(0,\pi,\pi )$, signaling again that the ADS phase is entered upon a band inversion in the anomalous gap that we verify to be the only gap with edge states in the spectrum for this termination in Fig. 8c. Turning to the *ZZ*_2_-edge, we see that the boundary states are determined by the paths along **b**_2_ and thus result in the same outcomes (Zak phases) as for the “arm chair” termination above, meaning that we predict edge states in the anomalous gap, which we confirm in Fig. 8c. Finally, when we consider the *ZZ*_3_-edge, the boundary spectrum is determined by paths along the direction **v** = − (**b**_1_−**b**_2_). We then obtain $({\gamma }_{{{{{{{{{\mathcal{B}}}}}}}}}_{1}}^{AL}[{{{{{{{\bf{v}}}}}}}}],\,{\gamma }_{{{{{{{{{\mathcal{B}}}}}}}}}_{2}}^{AL}[{{{{{{{\bf{v}}}}}}}}],\,{\gamma }_{{{{{{{{{\mathcal{B}}}}}}}}}_{3}}^{AL}[{{{{{{{\bf{v}}}}}}}}])=({\gamma }_{{{{{{{{{\mathcal{B}}}}}}}}}_{1}}^{ADS}[{{{{{{{\bf{v}}}}}}}}],\,{\gamma }_{{{{{{{{{\mathcal{B}}}}}}}}}_{2}}^{ADS}[{{{{{{{\bf{v}}}}}}}}],\,{\gamma }_{{{{{{{{{\mathcal{B}}}}}}}}}_{3}}^{ADS}[{{{{{{{\bf{v}}}}}}}}])=(\pi,0,\pi )$. Consistent with the interpretation that the relevant non-contractible paths in the **v**-direction do not cross the string of the anomalous gap, no edge states are anticipated for this termination. This is reflected in the similarity of Zak phases for both the ADS phase and AL and corroborated by our numerical results that show no edge states in either of the gaps of our system, see Fig. 8c. We finally note that this explicit evaluation sets the stage for more types of Anomalous Dirac String phases. One may, for example, consider a system that has edge states in the anomalous gap as well as the other gaps. We therefore believe that our results mark an important stepping stone for future pursuits.

## Data Availability

All data and descriptions of codes accompanying this publication are directly available within the publication.
